# Unilateral Anterior Scleritis Following the Booster Shot of Inactivated COVID-19 (Sinopharm) Vaccine in a 52-Year-Old Woman: A Case Report

**DOI:** 10.1155/crrh/6614757

**Published:** 2024-12-28

**Authors:** Kimia Jazi, Mahnaz Rahimi, Fatemeh Hasani, Maryam Shirmohammadi, Maryam Masoumi

**Affiliations:** ^1^Student Research Committee, Faculty of Medicine, Qom University of Medical Sciences, Qom, Iran; ^2^Golestan Research Center of Gastroenterology and Hepatology, Golestan University of Medical Sciences, Gorgan, Iran; ^3^Clinical Research of Development Unit, Shahid Beheshti Hospital, Qom University of Medical Sciences, Qom, Iran

**Keywords:** adverse events, case report, COVID-19, COVID-19 vaccines, scleritis

## Abstract

The only way to mitigate the spread of coronavirus disease 2019 (COVID-19) pandemic was vaccines. While effective in decreasing the rate and severity of the disease, there also have been considerable adverse events. Since the birth of vaccines, adverse reactions accompanied the immunity, and COVID-19 vaccines are no exceptions. This is a report about a 52-year-old female patient who presented with bilateral redness of the eyes, with normal bilateral visual acuity, postbooster dose of the Sinopharm COVID-19 vaccine. She had no significant past history of any disease or any similar reactions after previous doses. All her physical examinations were normal. Ophthalmic examination disclosed diffuse erythema, and mild scleral edema consistent with bilateral anterior diffused scleritis with negative phenylephrine test. Thereafter, with a course of tapering doses of prednisolone (30 mg at the onset) combined with azathioprine (100 mg/day), over a 2-week period, the condition completely resolved. Very few vaccination-related adverse events may manifest an unrecognized underlying autoimmune vasculopathy which may also require urgent management. As in this case, ocular adverse events, as highlighted, are highly associated with undiagnosed autoimmune diseases and therefore warrant careful assessment by clinicians.

## 1. Introduction

In December 2019, the world faced the outbreak of coronavirus disease 2019, known as COVID-19, of which the severe acute respiratory syndrome coronavirus 2 was known as the causative pathogen. When the virus started to spread all over the world, in the beginning of March 2020, WHO officially declared the disease as a pandemic [1]. Inevitably, mass vaccination became the only way to prevent and control the unleashed pandemic [2]. Within about 2 years, 155 vaccine candidates were developed, of which 23 were authorized, following different strategies: inactivated, mRNA, viral vector, nanoparticle-based peptide vaccines, etc. All authorized vaccines have shown promising efficacy, but the adverse events (AEs) and serious AEs (SAEs) remained an unknown challenge [3]. The most common AEs were injection site pain or tenderness, fatigue, headache, rash, fever, chill, as well as myalgia, and arthralgia [4]. Moreover, thrombosis and thrombocytopenia, myocarditis or pericarditis, inflammatory myositis, and autoimmune diseases were frequently reported SAEs [4]. The exact mechanism remains vague; however, immune cross-reactivity by antibodies produced after vaccination, bystander activation of T cells which is also responsible for rheumatic autoimmune diseases, adjuvants, and even additive substances to preserve the vaccine could all induce autoreactivity [5]. To date, few studies have reported cases of ocular inflammatory AEs after the first or second dose of vaccination, including white dot syndrome, pan uveitis, choroiditis, along with scleritis and episcleritis [6]. More importantly, not only approach to SAEs is important but also they can masquerade as connective tissue diseases highlighting that late intervention could result in eye lost.

Herein, we reported a case of 52-year-old woman presented with simple anterior scleritis following third dose of Sinopharm COVID-19 vaccination.

## 2. Case Presentation

**Figure 1:**
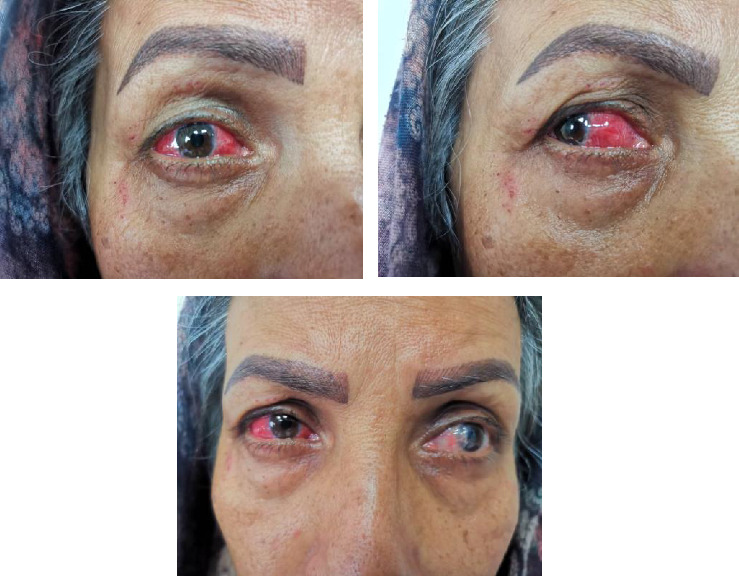
Unilateral anterior scleritis following COVID-19 Sinopharm vaccination 3 days after the third dose.

**Figure 2:**
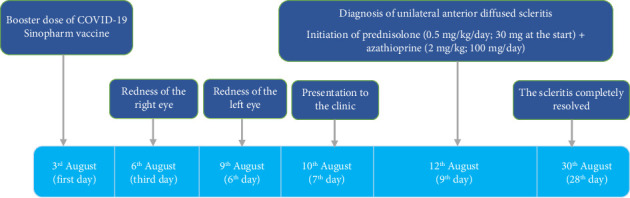
Timeline of symptoms from the beginning to follow-up sessions.

A 52-year-old woman presented with complete redness in the right and a slight color change in the left eye for 1 week (Figure 1). She had no history of hypersensitivity reaction or similar events. A week ago, she was vaccinated with the third dose of COVID-19 vaccine. The patient first developed right eye redness 3 days after the third dose of inactivated Sinopharm vaccine (BBIBP-CorV), which was slightly spread to the left side 3 days after. She presented to our clinic in the seventh day after her eye redness (Figure 2). No history of familial vasculopathies or recent trauma to the eyes was detected. Moreover, on the first and second dose of vaccination, both Sinopharm, a year and 6 months before, in respect, she experienced no similar symptoms or infrequent AEs. Initially, she was prescribed a corticosteroid eye drop immediately after following the left eye erythema (on the sixth day after vaccination), causing no change in her condition (Figure 2). On physical examination, the vital signs of the patient were within normal limits and stable with no respiratory distress and fever. No history of previous fever and chills, night sweats, or weight changes were detected. No signs of lymphadenopathy or splenomegaly were detected. The neurological examination of the patient was unremarkable. Ophthalmic examination revealed no signs and symptoms of eye discharge, pain, photophobia, and itching. She had no exophthalmia or proptosis. Besides, her past medical history included pterygium on her left eye conjunctiva during the past year, which was well-controlled. According to the oculist, her previous visual acuity was reported to be 20/20 bilaterally before the presence of redness and the same in the presence of redness. Wide spread erythema and slight scleral edema were present only in the right eye. Intraocular pressures were reported 17 and 16 mmHg in the right and left eye, respectively. Fundus examination was normal in both eyes. Further slit lamp examination showed crisscrossed inflamed scleral vessels that were adherent to sclera in the right eye. The globe was tender to touch, particularly at right side, and phenylephrine test result was negative bilaterally. There was no sign of inflammation, scleral cells or injection, or necrosis bilaterally. Microvascular dilatation was seen in various layers in conjunctiva, and also deep sclera at 3 and 9′o clock was seen at right. The examination of anterior and posterior chambers was unremarkable in both eyes. Additionally, there was no nodule, limbal phlycten, or AC cells. All these findings were compatible with unilateral anterior scleritis of the right eye.

Further tests were conducted considering the infectious causes, autoimmune diseases, systemic inflammatory disorders such as vasculopathies, and connective tissue diseases. Laboratory results showed an increase in c-reactive protein and erythrocyte sedimentation rate to 9.1 (positive > 9) and 39 (positive > 30), respectively. Moreover, all tests of liver function, kidney function tests, albumin, total protein, antineutrophil cytoplasmic antibodies (P-ANCA), perinuclear antineutrophil cytoplasmic antibodies (C-ANCA), fluorescent antinuclear antibody (FANA), complement 3 (C3), C4, anti-double-stranded DNA (anti-dsDNA), serology tests for hepatitis C virus (HCV), hepatitis B virus (HBV), and human immunodeficiency virus (HIV) tests were all negative or normal (Table 1). Also, the findings of stool examination and urinalysis did not reveal any findings in favor of renal disorders or infectious diseases. Radiological evaluation with chest X-ray (CXR) and computed tomography scan did not reveal any remarkable findings. Echocardiography and electrocardiogram showed no abnormal findings without any systolic or diastolic dysfunction and with normal ejection fraction. Electromyography-nerve conduction velocity (EMG-NCV) was normal.

By merging all the information obtained from the patient's symptoms and clinical assessments, autoimmune diseases, systemic inflammatory disorders such as vasculopathies, as well as connective tissue diseases other than infectious causes have all been excluded. The lack of other symptoms or any history of musculoskeletal or mucocutaneous involvement and normal EMG-NCV, in addition to normal rheumatological examination and lab data, rules out connective tissue disease or vasculopathies. Infection was rendered less likely by negative viral tests, normal CXR, and the absence of further manifestations of infectious diseases such as fever, weight loss, gastrointestinal, respiratory, or genitourinary involvements. Normal tests for all the other underlying causes, according to the recent COVID-19 vaccine, scleritis as an autoimmune reaction induced by Sinopharm COVID-19 vaccination was approved. For anterior, nonnecrotizing, noninfectious scleritis, nonsteroid anti-inflammatory drugs (NSAIDs), or topical steroids would be a great choice. However, she presented with a progressive scleritis with no response to home analgesic intake, and ophthalmic steroid usage in 4 days. Thus, to control the episode, a tapering dose of prednisolone (0.5mg/kg; starting with 30 mg/day) and azathioprine (2 mg/kg; 100 mg/day) was started as first-line and steroid-sparing immunosuppressive therapy, respectively.

After 2 weeks of follow-up, the scleritis was completely resolved, without any other complications. We continued azathioprine (100 mg/day) therapy for a year. During the next year, she was assessed three times for further signs or symptoms of autoimmune diseases, laboratory data on related autoantibodies, and slit lamp examination. All were normal, and the scleritis completely revealed with no further organ involvement.

## 3. Discussion

In this report, we present a case of scleritis after the third dose of COVID-19 vaccination with inactivated Sinopharm vaccine. Recently, Pichi and colleagues reported four cases of scleritis and episcleritis following the first dose of COVID-19 Sinopharm vaccination [7]. There also have been few reports of mild scleritis or episcleritis caused by live virus vaccination previously [8–11].

Scleritis is a severe inflammatory eye disease, which may present unilaterally or bilaterally, and is differentiated into posterior and anterior involvement with diffuse, nodular, and necrotizing forms. The inflammation may be idiopathic or secondary to infections, malignancies, autoimmune diseases responsible for at least 50% of cases, vasculopathies, trauma, or surgically induced, and medication side effects [12]. In this case, we first investigated the patient for a possible rheumatologic underlying cause. She did not have any history of skeletal involvement; furthermore, all the laboratory tests were within normal limits. Besides, she did not have signs and symptoms related to infectious disease or B symptoms related to malignancies. After ruling out all the possible causes, she was diagnosed with COVID-19 vaccination-induced scleritis. Testi et al. reported different ocular complications of COVID-19 vaccination [13]. In another report, none out of 7 patients (10%) with scleritis were vaccinated with Sinopharm. Moreover, to our knowledge, all the reports underlined that the first scleritis manifestations initiated after the first and second dose. Pichie et al. reported an anterior scleritis after the first dose of COVID-19 inactivated vaccination in a patient with history of rheumatoid arthritis, well controlled with topical steroid after a week [7]. Similar to our case, the participant had no other involvements in slit lamp examination; however, the vaccine complication was induced after the first dose. There are handful reports of anterior scleritis after Sinopharm injection, let alone after the booster dose with no previous complications [7–11, 14, 15]. Further investigations should clarify whether booster doses could induce more new additional immune reactions.

Although various reports of ocular AEs developed after COVID-19 vaccination, the pathogenesis and mechanism of this immune response remain the question. There have been certain studies demonstrating an association between COVID-19 vaccination and an increased risk of autoimmune diseases or related adverse reactions, such as autoimmune hepatitis, autoimmune glomerulitis, autoimmune rheumatism, and autoimmune scleritis [16–18]. The most frequently proposed mechanism includes molecular mimicry between scleral and vaccine peptides as well as hypersensitivity due to antigen-specific cell and antibody reactions [19]. Through cross-reactivity, the immune system falsely targets human proteins which trigger autoimmune reactions [20]. Kanduc et al., also showed same heptapeptides between the SARS-CoV-2 spike glycoprotein and human proteins [21]. Moreover, although safe in most of the population, vaccine adjuvants that were added to achieve the desired protection led to autoinflammatory syndromes particularly connective tissue disorders due to different nucleic acid metabolism [22]. Adjuvants could mimic toll-like receptor (TLR) ligands and stimulate immune responses [23]. The addition of alum as an adjuvant also aggravated immunopathologic reactions [24]. Noteworthy, inactivated COVID-19 vaccines stimulate T helper 2 cell reactions causing inflammation. In vaccines with adjuvants, T cells could be activated through bystander pathways. The auto-reactivated immune cells, migrate to inflammation site, produce cytokines and induce autoimmunity [5]. We recommend that further longitudinal large population studies investigate risk factors of post COVID-19 vaccination autoimmune reactions, to elucidate the need for further restrictions in patients with autoimmune disease or even high-risk healthy population.

The genes for immunity, inflammation, and coagulation are part of X chromosome, so we may suspect that viral interactions associated with human genes could induce an abnormal immune response in COVID-19. Besides, according to Manzo et al., the presence of excess antigen and the formation of relatively resistant soluble antigen-antibody immune complexes after exposure to SARS-CoV-2 may cause persistent inflammation in organs [25]. There are several reported cases of ocular inflammation and related conditions following COVID-19 vaccination. These include anterior uveitis, scleritis, episcleritis [6], multiple evanescent white dot syndrome, Vogt–Koyanagi–Harada disease [26], panuveitis [27], choroiditis [28], and central serous chorioretinopathy [29]. Most cases were successfully treated with corticosteroid therapy, including topical, intravitreal, and/or systemic administration, and many patients achieved complete recovery of their baseline visual acuity. A case series of orbital inflammation following mRNA vaccines was also described, successfully treated with oral prednisolone [30]. It is important for healthcare providers to be aware of these potential ocular reactions to COVID-19 vaccination and to monitor patients closely for any signs or symptoms of ocular inflammation or related conditions.

As mentioned, our patient did not show any serious reaction to previous doses of inoculation until the first booster. These reactions were found to be induced by activation of the secondary immune response, the memory cells [31]. Comparing to the first and second doses of vaccinations, Rahmani et al. reported that booster doses are more probable to stimulate rare AEs including neurological symptoms [32]. Moreover, authors suggested hormonal, genetic, and behavioral factors along with the time between the primary cycle to the first booster dose. The more the time between the booster dose and the first administration, the higher the immunogenic effect after the third shot [32]. Consequently, further studies could elucidate the proper time of the booster inoculations, particularly for high-risk patients in order to prevent serious reactions. Future investigations could also assess long-term effects of COVID-19 vaccination on ocular health, especially in relation to autoimmune conditions like scleritis.

Clinicians should always be aware of possible AEs of vaccines, even inactivated types such as Sinopharm. Autoimmune reactions should always be considered; however, before reaching the diagnosis, all further potential and life-threatening causes must be excluded. All organ functions should also be investigated, and autoimmunity is not limited to one side or even one time. Thus, patients with autoimmune reactions are susceptible to further involvements, and long-term follow-up is necessary.

This study had some limitations. We did not evaluate her ocular symptoms with magnetic resonance imaging (MRI) for possible further involvements. In addition, follow-up more than 1 year could also be valuable in this case. Unfortunately, we did not have access to slit lamp and IOP records.

## Figures and Tables

**Table 1:** Laboratory test results.

	Test, unit	Result	Reference range
Blood biochemistry	AST, U/L	17	Up to 35
	ALT, U/L	15	Up to 45
	Uric acid, mg/dL	4.3	Male 3.4–7
			Female 2.4–5.7

Hematology	WBC, μL	8800	4000–11000
	RBC, 10^6^/μL	4.84	4.2–6.3
	Hb, g/dL	12.6	12–16
	Hematocrit, %	40.5	30–45
	MCV, fL	83.67	80–100
	MCH, pg	26.03	27–32
	MCHC, g/dL	31.11	33–38
	Platelet, μL	363,000	150,000–450,000
	Neutrophil, %	40%	—
	Lymphocyte, %	25%	—
	Urine 24 h/Pr, mg/24 h	147	24–141
	Urine 24 h/Volume, g/24 h	1200	—
	Urine 24 h/Cr, mg/24 h	624	600–1800

Serology	ESR 1 h	39	Positive: > 30
	CRP quantitative	9.1	Positive: > 6
	Anti-B2-GLP1 antibody (IgG)	7	Positive: > 20
	Anti-B2-GLP1 antibody (IgM)	1.7	Positive: > 20
	ACA IgG	24	Positive: ≥ 12
	ACA IgM	1.5	Positive: ≥ 12
	LA antibody (dRVVT)	34	Direct: 25–45
	LA antibody (aPTT)	30	After mixing: 25–45
	Anti-ds DNA	6.6	Positive:≥ 100
	C3	165	90–180
	C4	54	10–40
	CH50	90	Positive:≥ 90
	FANA	Negative	Up to 1/100
	C-ANCA	Negative	Up to 1/10
	P-ANCA	Negative	Up to 1/10
	Anti-Sm/RNP antibody	0.1	Up to 20
	HLA-B27	Negative	—
	HLA-B5	Positive	—
	HLA-B51	Negative	—

Abbreviations: ACA, anticardiolipin antibody; ALT, alanine transaminase; anti-B2-GLP1 antibody, anti-b2-glycoprotein antibody; anti-dsDNA, anti-double-stranded DNA; anti-Sm/RNP antibody, anti-Smith/antinuclear ribonucleoprotein antibody; aPPT, activated partial prothrombin time; AST, aspartate aminotransferase; C3/C4, complement 3/4; CANCA, antineutrophil cytoplasmic antibodies; CH50, total hemolytic complement; CRP, c-reactive protein; dRVVT, diluted Russell viper venom time; ESR 1 h, erythrocyte sedimentation rate in one hour; FANA, fluorescent antinuclear antibody; Hb, hemoglobin; HBV, hepatitis B virus; HCV, hepatitis C virus; HIV, human immunodeficiency virus; HLA, human leukocyte antigen; LA antibody, lupus anticoagulant; MCH, mean corpuscular hemoglobin; MCHC, mean corpuscular hemoglobin concentration; MCV, mean corpuscular volume; PANCA, perinuclear antineutrophil cytoplasmic antibodies; RBC, red blood cell; WBC, white blood cell.

## Data Availability

The data that support the findings of this study are available on request from the corresponding author. The data are not publicly available due to their containing information that could compromise the privacy of research participants.
